# Relationships between quality of life and family function in caregiver

**DOI:** 10.1186/1471-2296-12-19

**Published:** 2011-04-15

**Authors:** Emiliano Rodríguez-Sánchez, Aníbal Pérez-Peñaranda, Andrés Losada-Baltar, Diana Pérez-Arechaederra, Manuel Á Gómez-Marcos, Maria C Patino-Alonso, Luís García-Ortiz

**Affiliations:** 1 Unidad de investigación del Centro de Salud de La Alamedilla. Salamanca. España; 2 Unidad de Investigación de Desarrollo Humano (UNIDESH). Universidad de Carabobo. Maracay. Venezuela; 3 Departamento de Psicología, Facultad de Ciencias de la Salud, Universidad ReyJuan Carlos, Alcorcón (Madrid), Spain; 4 Departamento de Estadística. Universidad de Salamanca. Salamanca. España

## Abstract

**Background:**

There are caregivers who see their quality of life (QoL) impaired due to the demands of their caregiving tasks, while others manage to adapt and overcome the crises successfully. The influence of the family function in the main caregiver's situation has not been the subject of much evaluation. The aim of this study is to analyse the relationship between the functionality of the family and the QoL of caregivers of dependent relatives.

**Methods:**

We conducted a cross-sectional study including 153 caregivers. Setting: Two health centers in the city of Salamanca(Spain). Caregiver variables analysed: demographic characteristics, care recipient features; family functionality (Family APGAR-Q) and QoL (Ruiz-Baca-Q) perceived by the caregiver. Five multiple regressions are performed considering global QoL and each of the four QoL dimensions as dependent variables. The Canonical Correspondence Analysis (CCA) was used to study the influence of the family function questionnaire on QoL.

**Results:**

Family function is the only one of the variables evaluated that presented an association both with global QoL and with each of the four individual dimensions (p < 0.05). Using the CCA, we found that the physical and mental well-being dimensions are the ones which present a closer relationship with family functionality, while social support is the quality dimension that is least influenced by the Family APGAR-Q.

**Conclusion:**

We find an association between family functionality and the caregiver's QoL. This relation holds for both the global measure of QoL and each of its four individual dimensions.

## Background

Disease and incapacity are common experiences that represent one of the greatest challenges for families, since the psychosocial problems occasioned by a person with dependence have an impact on the whole family system. In these family contexts many families suffer a deterioration in their quality of life (QoL), while others manage to adapt and overcome the crisis successfully [1-4].

Functional families are those in which the roles of all the members are laid down without critical points of assumed debilities and without positions of either artificial or assumed supremacy being held by any of the members and in which they all participate, work, contribute and cooperate on an equal basis and with enthusiasm for the collective welfare. Sometimes several members of the family take care of the dependent relative, but it is more common for the burden of the care to lie with a single person: the main caregiver [1,5,6]. This care affects the caregiver significantly in physical, mental, social and economic aspects. It produces an overload of tasks and it usually changes the functional dynamics of the family [6-9].

Although at this time there is a movement aimed at the study of the patient's quality of life, it underscores the importance of the caregiver's view [10], the fact of the matter is that is important to consider the quality of life of the family members responsible for the care of patients in a situation of dependence as to consider the QoL of the dependent himself/herself [1,4]. Quality of life is a global health indicator that provides information not supplied in the clinical instruments normally used, giving information on the physical, psychological and social dimensions of people's life [1,4,11,12].

The aim of this study is to analyse the influence of the functionality of the family in the QoL of the caregivers of family dependents and to determine the variables related to both dimensions.

## Methods

We conducted a cross-sectional study in two urban primary care health centres. Health professionals of both centres contacted 174 caregivers who provided their dependent relatives the main assistance for their basic daily life activities (BDLA). Those persons who did not share their place of residence with the relative, or who were impossible to locate either due to a change of address or being admitted to hospital or a nursing home, or on account of the patient's death, were excluded. At the end 153 caregivers were included.

The study was approved by the research ethics committee from health area of Salamanca, complies with Spanish data protection law 15/1999 and its recently developed specifications (Royal Decree (RD) 1720/2007) and all the subjects who took part in the study signed the informed consent form prior to their inclusion.

### Measures

The data were collected by means of a home interview to the caregivers and the dependent relatives by a psychologist and two nurses with prior training to apply the questionnaires. Variables related to the caregiver: a) Demographic characteristics (age, gender, marital status, educational level, occupation, relationship, age at the start of caregiving), hours of attention per day and months of caregiver. b) Family functionality perceived by the caregiver was evaluated with the Family APGAR Questionnaire (APGARq) validated in Spain [13]. This questionnaire rates satisfaction with family relations and distinguish five components of the family function: adaptability, partnership, growth, affection and resolve. It consists in five questions shown in Table 1, with three possible answers: 0 ("hardly ever"), 1 ("sometimes"), 2 ("always"). The total score range varies from 0 to 10, meaning the higher total score, the better family functioning. A global score of 7 points or more indicates family functionality, while a score of less than 7 points indicates family dysfunction. The internal consistency of this questionnaire in this study was 0.77. c) Quality of life was evaluated with Ruiz-Baca's Questionnaire (1993) [14]. This is made up of 39 items each with a Likert-type five-point scale comprising four dimensions: social support, general satisfaction, physical/mental well-being, work overload and free time (Cronbach's alpha = 0.94). Variables related to the dependent relative: a) Demographic characteristics (age, gender, education.); b) Cognitive status: the possible existence of cognitive deterioration in the care recipient is assessed with Pfeiffer's Test (Cronbach's alpha = 0.94) [15]. c) The functional capacity of the patients was assessed with Barthel's index [16], which evaluates the person's capacity to perform different BDLA (Cronbach's alpha = 0.91).

**Table 1:** Matrix used for the Canonical Correspondence Analysis (ACC) (Ter Braak, 1988)

QUALITY OF LIFE ÍTEMS: DIMENSIONS OF THE PERCEIVED QUALITY OF LIFE AND CARE OF PATIENTS QUESTIONAIRE (RUíZ Y BACA, 1993)	FAMILY ASSESSMENT ÍTEMS: FAMILY APGAR ITEMS
• Social support • General Satisfaction • Physical and Mental Wellness • Lack of work overload/free time	**SAF**: Are you satisfied with the help that you received from your family? **CPF**: Do you talk with your family about your household problems? **SQF**: Do you feel that your family loves you? **STF**: Are you satisfied with the time that you and your family share together? **DIF**: Do you discuss with your family about important decisions that affect the whole family?

### Analysis

We performed a chi-square test to establish the relation between independent qualitative variables, Student's t-test to evaluate the relation between two-category qualitative and quantitative variables, and correlation for the quantitative variables. Five multiple regressions were performed, considering the global QoL and each of the four dimensions of the QoL questionnaire (social support, general satisfaction, physical/mental well-being, and absence of work overload/free time). In each of the analyses the same explanatory variables are used for the caregiver (caregiver's gender, occupation and age, age at the start of the caregiving, care hours/day, caregiver's time, caregiver's educational level) and for the dependent relative (gender, age and educational level). While the caregiver's gender and the patient's gender remain fixed in the regression model, the stepwise method is applied to the rest of the variables. In order to study the influence that each item of the APGARq has on the QoL of the caregivers, the Canonical Correspondence Analysis (CCA), proposed by Braak, was used [17]. The starting point is two matrices, one containing the information for the 153 subjects under study, relating to the items of the dimensions of the Ruíz-Baca Questionnaire QoL, and a second matrix containing the information of the APGARq items relating to the family function (Table 1). The family function items are represented by vectors, which are constructed by joining the point represented by the item with the centre of gravity of the HyperCloud projected on the subspace of maximum inertia. The angle that the respective items evaluating the different aspects of the family function form between one another allows us to estimate the degree of covariation between the different aspects. To evaluate the influence that a given item of family function has on each of the aspects of QoL, we merely draw the perpendicular to the vector joining the family function item with the origin of coordinates. The points representing the different aspects of QoL whose projection of the family function variable vector lie closer to the tip of the arrow tell us which have higher values in relation to this variable. Accepting an alpha risk of 0.05 and a beta risk of 0.05 in a two-sided test, with 46 subjects in first group (dysfunctional family) and 107 in the second (functional family) and common standard deviation of 0.67 is enough to recognize as statistically significant a difference greater or equal than 0.43 units of mean global QoL. The program used for the processing and analysis of the data was SPSS/PC+ (V.15.0), except for the CCA, where the Canoco 4.5 for Windows package was used [18-20].

## Results

Sociodemographic data for the resulting sample and the descriptive statistics for the assessed variables are shown in Table 2. In Table 3 we observe the APGARq results, with a global mean score of 1.52 (scale 0-2), with the highest rated item being "feels that his/her family loves him/her" (SQF)(1.78). A 69.93% of the families were in a situation of normal functionality (≥ 7 points).

**Table 2:** Demographic characteristics of the caregivers and of the patients studied

**Caregivers**		**Dependent Patients**	
**Age (years)**		**Age (years)**	
Global (n = 153)	63.8 ± 12.8	Global (n = 153)	79.14 ± 17.3
Males (n = 42)	66.8 ± 13.6	Males (n = 48)	74.90 ± 19
Females (n = 111)	62.7 ±12.4	Females (n = 105	81.08 ± 15.9
Age when they started being caregivers	53.0 ± 13.9		
Hours per day caring for	19.2 (7.2)		

**Marital status**		**Level of functional dependence **^**a**^	
Married	99 (64.7%)	Total	76 (49.7%)
Single	42 (27.5%)	Severe	28 (18.3%)
Widow	12 (7.8%)	Moderate	32 (20.9%)
		Slight	17 (11%)

**Level of Education**		**Level of Education**	
Graduate	25 (16.3%)	Graduate	0 (0.0%)
Secondary studies	29 (19.0%)	Secondary studies	11 (7.2%)
Primary studies	88 (57.5%)	Primary Studies	107 (69.9%)
Illiterate	11 (7.2%)	Illiterate	20 (13.1%)
Non answered	0 (0.0%)	Non answered	15 (9.8%)

**Relationship with the dependent**		**Cognitive damage**^**b**^	
Son/daughter	72 (47.1%)	Important	63 (41.2%)
Spouse	41 (26.8%)	Moderate	26 (17.0%)
Parents	22 (14.4%)	Slight	14 (9.2%)
Others	18 (11.9%)	Without damage	50 (32.7%)

**Activities**:			
Housewife and caregiver	115(75.2%)		
Worker and caregiver	33(21.6%)		
Other	5(3.3%)		

The data are presented as the Mean ± Standard Deviation or as number and percentage.

a.- Assessment of the level of functional dependence according to Barthel categorization: Total dependence: Value 0-20; Severe dependence: 21-60; Moderate dependence: 61-90; Slight dependence: 91-99; Independent: 100.

b.-Cognitive damage assessed with Pfeiffer's measure: Severe damage: 8-10 errors; Moderate: 5-7; Slight: 3-4; Without damage: 0-3.

**Table 3:** Family functioning assessment with the Family-APGAR and quality of life assessment with Ruiz and Baca's questionnaire

**Family APGAR Questionnaire**^**a**^	**Mean**	**±SD**
**Family function APGAR (global mean)**	**1.52**	**0.46**

1. Are you satisfied with the help that you received from your family?	1.42	0.67
2. Do you talk with your family about your household problems?	1.49	0.61
3. Do you feel that your family loves you?	1.78	0.49
4. Are you satisfied with the time that you and your family share together?	1.50	0.64
5. Do you discuss with your family about important decisions that affect the whole family?	1.41	0.74

**Caregivers with a functional family **^**b **^**n (%)**	**107 (69.93%)**	

		
**Quality of life dimensions **^**c**^	**Mean**	**±SD**

**Global perceived quality of life**	**3.25**	**0.67**
Social support	3.63	0.68
General satisfaction	3.14	0.74
Physical and mental wellness	3.13	1.05
Lack of work overload/free time	2.78	1.04

Non significant differences between males and females.

Data showed as mean ± standard deviation or as number and percentages.

^**a **^Family APGAR valid values are between 0 and 2.

^**b **^Total value between 0-10: ≥ 7 Family function; <7: Family dysfunction.

^**c **^Valid values for the Ruiz y Baca questionnaire are from 1 to 5, higher punctuation means better quality of life.

The global QoL score obtained for the total sample was 3.25 (scale 1-5). The normofunctional families have a QoL mean score of 3.84 (SD: 0.60) and the dysfunctional ones 2.87 (SD: 0.65) (p < 0.05). In its multidimensional character, QoL showed the best score in perceived social support (3.63), whereas work overload and lack of free time (2.78) were the dimensions that showed greater deterioration.

### Multiple linear regression analysis of QoL

In Table 4 we present the variables included in the equation Global QoL and its dimensions as dependent variables, adjusted by caregiver's and the patient's gender. In the multiple linear regression, taking the global QoL as the dependent variable, family function (beta = 0.139) and to be spouse (beta = -0.461) remained in the equation. In the multiple linear regression of the different dimensions of quality of life, the family function remained always in the equation. In the social support dimension, to be a grandchild (beta = 0.857) and a son/daughter (beta = 0.464) remained also in the equation; and the lack of overload dimension remained to be spouse (beta = -0.607), cognitive deterioration (beta = 0.367) and level of functional dependence (beta = -0.409).

**Table 4:** Multiple linear regression of the global quality of life and its dimensions as dependent variable (Beta values)

	**Dependent Variable**:				
**Independent Variables**:	**GLOBAL QOL**	**SOCIAL SUPPORT**	**GENERAL SATIS-FACTION**	**PHYSICAL-MENTAL WELLNESS**	**LACK OF OVERLOAD**
	
**Related to the caregiver**:					
Sex	0.225	-0.027	0.104	0.594*	0.447*
Family Function	0.139 *	0.101 *	0.131 *	0.192 *	0.121 *
Relationship: spouse	-0.461 *	.	-0.448 *	-0.551 *	-0.607 *
Relationship: Son/daughter		0.464 *			
Relationship: Grandchild		0.857 *			
**Related to the dependent**:					
Sex	0.049	-0.110	0.142	0.176	-0.204
Cognitive damage (Pfeiffer Test)					0.376
Level of functional dependence (Barthel I.)					-0.409 *

QOL: quality of life

* p < 0.05

### Canonical Correspondence Analysis (CCA)

**Figure 1:**
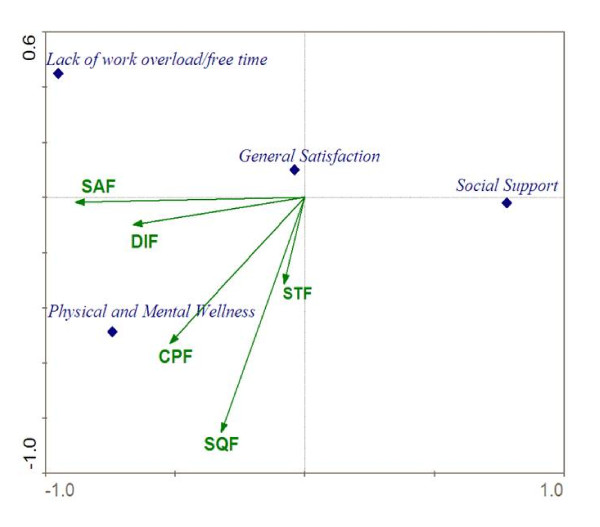
**Distribution diagram resulting from studying the influence of every APGAR questionnaire item on the caregivers' Quality of Life using Canonical Correspondence Analysis (CCA)**. SAF: Are you satisfied with the help that you received from your family?, CPF: Do you talk with your family about your household problems?, SQF: Do you feel that your family loves you?, STF: Are you satisfied with the time that you and your family share together?, DIF: Do you discuss with your family about important decisions that affect the whole family?

The ordination diagram for the influence that the individual items of the APGARq have on the quality of life of the caregivers, using **CCA**, can be seen in Figure 1. Special mention should be made regarding the small angle formed between the variables "Are you satisfied with the help you receive from your family?" (SAF) and "Are important decisions made jointly at home?" (DIF), which indicates a high correlation between these aspects. This may be interpreted as that those caregivers who make the important decisions with their family are satisfied with the help that they receive from them. For the items "Are you satisfied with the time that your family and you spend together?" (STF) and SQF, we found a similar interpretation (i.e. those who spend time with their families feel that their families love them). Furthermore, special mention should be made of the independence between items SAF and STF.

In Figure 1 we observe how the dimension relative to social support was the dimension that appears to be less influenced by the family function items. The dimension absence of work overload and free time marks the upper end of the gradient for the variable SAF, so we may interpret that those caregivers who have absence of work overload and free time are satisfied with the help that they receive from their family. In a similar way, we observe how, if we project the dimension relating to Physical and mental well-being on SAF, DIF, CPF, SQF and STF, it marks the upper end of the gradient in all of them. This means that for a caregiver to be able to have a good state of physical and mental well-being, he or she has to be satisfied with the help received from his or her family, converse with them, feel the affection of his or her family, be satisfied with the time that his or her family spend with him or her, and make family decisions.

## Discussion

This study shows the relationship between family functionality and the caregiver's QoL. According to these results, this relationship is found both for the global measure of QoL and for each of the four dimensions of this variable. While the dimension relating to physical and mental well-being is the one that presents a closer relationship with family functionality, social support is the dimension that seems to be less influenced by the family function items.

The Family APGAR test and the Ruiz-Baca QoL test results suggest that caregivers' perceived physical and mental well-being is related with being satisfied with the help received from their family, conversing with their relatives, feeling the affection of their family, being satisfied with the time that their family spends with them, and making family decisions. Providing support for caregivers or relieving the situations of work overload and stress that they are suffering may not br enough. These results indicate that it is important to ensure that when caregivers receive support, they actually perceive it like that, expressing their improvement at both the physical and emotional level.

There are caregivers of family dependents who present extremely different levels of QoL in similar circumstances [1-3]. The results of this study suggest that family function plays a particularly important role when it comes to explaining the QoL of the caregivers. This study adds to previous studies, in which the role of family functionality has been shown in the stress process in dementia patient caregivers [21,22], the significant capacity of this variable in explaining QoL. We consider that family function affects not only those who are caring for relatives with dementia, but also has an impact on caregivers of dependents with a wide range of medical conditions, like those included in this study.

In these study we found that the perception of QoL shown by caregivers differs according to the degree of kinship: only in children and grandchildren we found a positive correlation with social support [23]. This fact was even more significant upon observing that being the spouse correlates negatively both in global QoL and in the three dimensions other than social support. Neither family function nor QoL seem to have a significant correlation with the sociodemographic or educational level variables of caregivers according to the obtained results, which contrasts with findings from other studies [24,25]. Perhaps the many years of caregiving are associated with exhaustion, but in this study neither the years of care nor the age at the start of caregiving proved to be decisive in caregiver's quality of life, as was found by other authors [26].

Other studies have pointed out negative consequences of the two stressors analysed relating to the dependent person compared with such other consequences as overload and mental health [8,24,27]. However, with regard to QoL, our data showed that both stressors had practically no consequences. We did not found significant differences either in the QoL of caregivers in relation to age or gender of dependent persons. We therefore agree with other authors who contend that it would make more sense to talk of caregivers of family dependents in general (instead of caregivers of elderly persons or caregivers of dementia patients, etc.), as their emotional problems and vulnerability depend more on the caregiver's own abilities and resources than on the specific set of problems presented by the person being cared for [25-28]. In this respect, the results of this study suggest that family function is an important variable to be taken into account. A dysfunctional family represents a significant vulnerability factor which affects the caregiver's QoL as well as the quality of care. Chronic disease and, above all, dependence could affect the family and make it dysfunctional [1,29]. A negative characteristic of the dysfunctional family is that conflicts grow as communications decline or disappear entirely. The results of this study suggest that those caregivers who make important decisions together with their family are the ones who feel a better state of physical and mental well-being. These results support what has been found by other authors who state that when the decision to become a caregiver is taken on one's own initiative, the fact of being a caregiver may be associated with a greater probability for positive effects related to the care [27,30,31].

This study has some limitations. We should have in mind that we have not used tests specifically designed for family caregivers when evaluating both the QoL and the family function. However, we consider that, in order to analyse how the QoL of the relatives who have ceased to care for the relatives changes, general tests are preferable [32]. The fact of being a caregiver, unlike those with chronic diseases [2,3,12,23,25-28], for whom numerous specific instruments have been designed [30], is usually a transitory situation, and on the other hand, the caregivers are usually seniors with their own multimorbidities [26,33].

## Conclusions

This research suggest that caregivers' perceived family function is an important variable that should be taken into account in the assessment of the patient/caregiver situation when designing the care plan for both of them. In conclusion, we found an association between family functionality and caregivers' QoL. This relation holds for both for the global measure of QoL and for each of its four individual dimensions.

## Competing interests

The authors declare that they have no competing interests.

## Authors' contributions

Conception of the idea for the study: ERS and LGO. APP and DAP Development of the protocol, organization and funding, participated in the interview. MCP participated in the design of the study and performed the statistical analysis. ALB and MAG participated in its design and coordination and helped to draft the manuscript. Writing of the manuscript: ERS and LGO. All the authors have read the draft critically, to make contributions, and have approved the final text.

## Pre-publication history

The pre-publication history for this paper can be accessed here:

http://www.biomedcentral.com/1471-2296/12/19/prepub
